# Impact of Examined Lymph Node Count on Precise Staging and Long-Term Survival of Resected Non–Small-Cell Lung Cancer: A Population Study of the US SEER Database and a Chinese Multi-Institutional Registry

**DOI:** 10.1200/JCO.2016.67.5140

**Published:** 2016-12-27

**Authors:** Wenhua Liang, Jiaxi He, Yaxing Shen, Jianfei Shen, Qihua He, Jianrong Zhang, Gening Jiang, Qun Wang, Lunxu Liu, Shugeng Gao, Deruo Liu, Zheng Wang, Zhihua Zhu, Calvin S.H. Ng, Chia-chuan Liu, René Horsleben Petersen, Gaetano Rocco, Thomas D’Amico, Alessandro Brunelli, Haiquan Chen, Xiuyi Zhi, Bo Liu, Yixin Yang, Wensen Chen, Qian Zhou, Jianxing He

**Affiliations:** Wenhua Liang, Jiaxi He, Jianfei Shen, Qihua He, Jianrong Zhang, and Jianxing He, The First Affiliated Hospital of Guangzhou Medical University; Wenhua Liang, Jiaxi He, Jianfei Shen, Qihua He, Jianrong Zhang, and Jianxing He, Guangzhou Institute of Respiratory Disease & China State Key Laboratory of Respiratory Disease; Zhihua Zhu, Cancer Center of Sun Yat-Sen University; Qian Zhou, The First Affiliated Hospital of Sun Yat-Sen University, Guangzhou; Jianfei Shen, Taizhou Hospital of Zhejiang Province, Wenzhou Medical University, Linhai; Yaxing Shen and Qun Wang, Shanghai Zhongshan Hospital of Fudan University; Gening Jiang, Shanghai Pulmonary Hospital of Tongji University; Haiquan Chen, Fudan University Shanghai Cancer Center, Shanghai; Lunxu Liu, West China Hospital, Sichuan University, Chengdu; Shugeng Gao, Cancer Institute & Hospital, Chinese Academy of Medical Sciences; Shugeng Gao, Peking Union Medical College; Shugeng Gao, National Cancer Center; Deruo Liu, China and Japan Friendship Hospital; Xiuyi Zhi, Beijing Xuanwu Hospital of Capital Medical University; Bo Liu and Yixin Yang, Academy of Mathematics and Systems Science in the Chinese Academy of Sciences, Beijing; Zheng Wang, Shenzhen People’s Hospital, Shenzhen; Calvin S.H. Ng, The Chinese University of Hong Kong, Hong Kong, Special Administrative Region; Wensen Chen, Nanjing Medical University; Wensen Chen, the First Affiliated Hospital of Nanjing Medical University, Nanjing, People’s Republic of China; Chia-chuan Liu, Sun Yat-Sen Cancer Center, Taipei, Republic of China; René Horsleben Petersen, University Hospital of Copenhagen, Rigshospitalet, Copenhagen, Denmark; Gaetano Rocco, Istituto Nazionale Tumori Fondazione G. Pascale, Naples, Italy; Thomas D’Amico, Duke University Medical Center, Durham, NC; and Alessandro Brunelli, St James’s University Hospital, Leeds, United Kingdom.

## Abstract

**Purpose:**

We investigated the correlation between the number of examined lymph nodes (ELNs) and correct staging and long-term survival in non–small-cell lung cancer (NSCLC) by using large databases and determined the minimal threshold for the ELN count.

**Methods:**

Data from a Chinese multi-institutional registry and the US SEER database on stage I to IIIA resected NSCLC (2001 to 2008) were analyzed for the relationship between the ELN count and stage migration and overall survival (OS) by using multivariable models. The series of the mean positive LNs, odds ratios (ORs), and hazard ratios (HRs) were fitted with a LOWESS smoother, and the structural break points were determined by Chow test. The selected cut point was validated with the SEER 2009 cohort.

**Results:**

Although the distribution of ELN count differed between the Chinese registry (n = 5,706) and the SEER database (n = 38,806; median, 15 versus seven, respectively), both cohorts exhibited significantly proportional increases from N0 to N1 and N2 disease (SEER OR, 1.038; China OR, 1.012; both *P* < .001) and serial improvements in OS (N0 disease: SEER HR, 0.986; China HR, 0.981; both *P* < .001; N1 and N2 disease: SEER HR, 0.989; China HR, 0.984; both *P* < .001) as the ELN count increased after controlling for confounders. Cut point analysis showed a threshold ELN count of 16 in patients with declared node-negative disease, which were examined in the derivation cohorts (SEER 2001 to 2008 HR, 0.830; China HR, 0.738) and validated in the SEER 2009 cohort (HR, 0.837).

**Conclusion:**

A greater number of ELNs is associated with more-accurate node staging and better long-term survival of resected NSCLC. We recommend 16 ELNs as the cut point for evaluating the quality of LN examination or prognostic stratification postoperatively for patients with declared node-negative disease.

## INTRODUCTION

Lung cancer is the leading cause of cancer-related mortality worldwide, with approximately 85% of patients having non–small-cell lung cancer (NSCLC).^1^ For early-stage resectable NSCLC, radical surgical resection remains the standard of care. However, the postresection 5-year survival rate is only 50% to 60%.^2^

Patients with positive lymph node (LN) metastasis have a higher risk of disease recurrence; thus, LN involvement is one of the most important determinants for both prognosis and decisions about treatment strategy in patients with resectable NSCLC. LN sampling or dissection plays an important role in precise nodal staging by identifying LN involvement and determining the extent of disease and in the therapeutic effect of potential LN metastatic lesion clearance. Precise staging is the key to appropriate delivery of adjuvant therapies. For example, adjuvant chemotherapy is recommended for patients with NSCLC who have any sign of LN metastasis,^3^ and the benefits of adjuvant chemotherapy in patients with node-positive NSCLC have been well validated.^4^ In addition, the therapeutic effects of adjuvant therapy on any unsuspected residual disease may also contribute to recurrence risk reduction and improved survival.^5^

For many cancers, such as GI and breast cancers, the association between the greater number of examined LNs (ELNs) and improved patient survival has been well studied. Therefore, National Comprehensive Cancer Network (NCCN) guidelines recommend that the minimal number of LNs be removed or sampled for adequate nodal staging.^6-9^ However, the current NCCN guidelines for NSCLC recommend that surgeons sample only the LN stations and state that one or more nodes be sampled from all mediastinal stations (2R, 4R, 7, 8, and 9 for right-sided cancers; 4L, 5, 6, 7, 8, and 9 for left-sided cancers), according to the LN map from the International Association for the Study of Lung Cancer.^10^ The minimum number of LNs that should be examined to accurately stage or identify high-risk patients for disease recurrence has not yet been well established or emphasized in NSCLC.

Some studies have shown a correlation between the number of ELNs and long-term survival, but the results of these studies are contradictory.^11-18^ Studies that were based on US SEER data identified a positive correlation between the number of ELNs and improved overall survival (OS),^11-17^ whereas another study that was based on an Asian cohort suggested the reverse trend.^18^ Most of these studies were flawed because of the use of univariable analysis that was biased by other significant confounders. In addition, the impact of the ELN number on stage correction in NSCLC has not been studied. The diverse biologic behaviors that underlie various histologic types may greatly influence the role of LN examination in prognostication, but none of the studies examined the role of ELNs separately according to histology. Moreover, the methods for identifying the cut points were not sufficiently robust.^11-18^

To address these unresolved issues, we performed analyses of two large databases that include various regions, ethnicities, and clinical preferences, which may more accurately portray real-world conditions to further confirm the relationship between ELN and long-term survival and stage migration. We used a multivariable analysis to determine an optimal threshold for ELN count.

## METHODS

### Patient Population

#### Chinese multi-institutional registry.

A multi-institutional registry of consecutively collected data on patients with NSCLC who underwent surgical resection between January 2001 and December 2008 at the departments of thoracic surgery of seven institutions in the People’s Republic of China (The First Affiliated Hospital of Guangzhou Medical University; Shanghai Pulmonary Hospital of Tongji University; Shanghai Zhongshan Hospital of Fudan University; West China Hospital, Sichuan University; China and Japan Friendship Hospital; Shenzhen People’s Hospital; and Cancer Center of Sun Yat-Sen University) was used for the analyses. Data collection and processing have been detailed previously.^19^ Briefly, LNs were harvested during surgical resection of NSCLC, and the tissue was examined postoperatively by pathologists. The ELN counts in the registry were generated by adding the surgeons’ intraoperative harvested LN count to the number of LNs identified by pathologists postoperatively. Ethical approval was obtained from participating institutions through their respective institutional review boards. In cases in which individual patient consent was not identified, the chairperson of the ethics committee waived the need for patient consent. Patients were staged by using the seventh edition of the TNM classification. An independent cohort that comprised consecutive patients with NSCLC who underwent radical resection between August 2009 and December 2011 at The First Affiliated Hospital of Guangzhou Medical University was also used for cutoff validation.

#### SEER Database.

SEER is representative of the US population, with patient-level data abstracted from 18 geographically diverse populations that represent rural, urban, and regional populations. NSCLC cases between 2001 and 2008 (to match the time span of the Chinese cohort) in the SEER public access database and their corresponding details were retrieved with the use of SEER*Stat version 8.1.5 software. Patients were uniformly reviewed and staged according to the seventh edition of the TNM classification. An independent cohort of cases from 2009 was also retrieved for cutoff validation.

### Patient Selection

Patients who underwent surgical resection for first primary NSCLC with at least one examined LN were eligible. Patients with missing values on ELN count and clinical features were excluded. Patients with stage IIIB or IV disease were not eligible because surgery is not the standard of care. ELN count was collected from database records.

### Statistical Analyses

#### Multivariable regression analyses.

We used the χ^2^ test^20^ to compare differences between categorical variables and *t* test^21^ for continuous variables. The Cox proportional hazards regression model^22^ was used to determine the effect of ELN number on OS and to visualize the survival curves, which were adjusted for other significant prognostic factors. On the basis of the assumption that more ELNs present a greater opportunity to identify positive LNs, stage migration was assessed by correlating the ELN number and the proportion of each node stage category (node negative versus node positive) by using a binary logistic regression model^23^ after adjusting for other potential confounders associated with ELN number or node stage before or during surgery. All calculations were performed with SPSS version 17.0 for Windows software (IBM Corporation, Chicago, IL).

#### Accuracy of the number of involved LNs.

A mathematical model of the number of nodes examined was created by using hypergeometric distribution and Bayes theorem in accordance with the procedures previously described by Iyer et al.^24^ The resulting model was used to estimate the accuracy of the reported number of positive nodes, in particular, the probability of having at least one undetected positive LN among patients with node-negative disease with different observed sampling numbers.

#### Fitting of curves and determination of structural break points.

The curves of odds ratios (ORs; stage migration) and hazard ratios (HRs; OS) of each ELN count compared with one ELN (as a reference) as well as the curves of mean positive number and probability of undetected positive LNs were fitted by using a LOWESS smoother with a bandwidth of 2/3 (default) by using R version 3.2.2 software (Bell Laboratories, Murray Hill, NJ; https://cran.r-project.org/bin/windows/base/old/3.2.2).^25^ Structural break points were then determined by Chow test with the use of SAS 9.3 software (SAS Institute, Cary, NC).^26^ The break points were considered the threshold of clinical impact. *P* < .05 was considered statistically significant.

## RESULTS

### Patient Characteristics and Distribution of ELN Number

**Fig 1.**
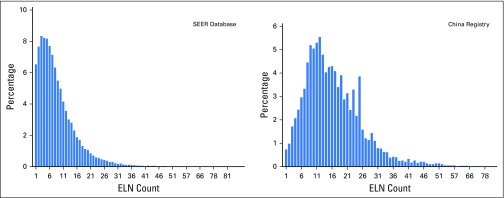
Distribution of the number of harvested lymph nodes in the China registry and the SEER database. ELN, examined lymph node.

A total of 38,806 patients in the US SEER cohort and 5,705 patients in the Chinese cohort who met the eligibility criteria were included in this study. The baseline characteristics of each cohort are listed in Table 1. There were 1,897 deaths recorded (censored, 66.7%) during a median follow-up of 3.6 years in the Chinese cohort, and 17,029 deaths were recorded (censored, 56.1%) during a median follow-up of 5.0 years in the SEER cohort. The distribution of ELN number (Fig 1) differed between the cohorts; the Chinese cohort had a larger number of ELNs (median, 15; interquartile range, 10 to 22) than the SEER cohort (median, 7; interquartile range, 4 to 12). An independent cohort with eligible cases from the SEER database in 2009 as well as another cohort from The First Affiliated Hospital of Guangzhou Medical University between 2009 and 2011 were retrieved for validation of the cut point (Table 1).

**Table 1.**
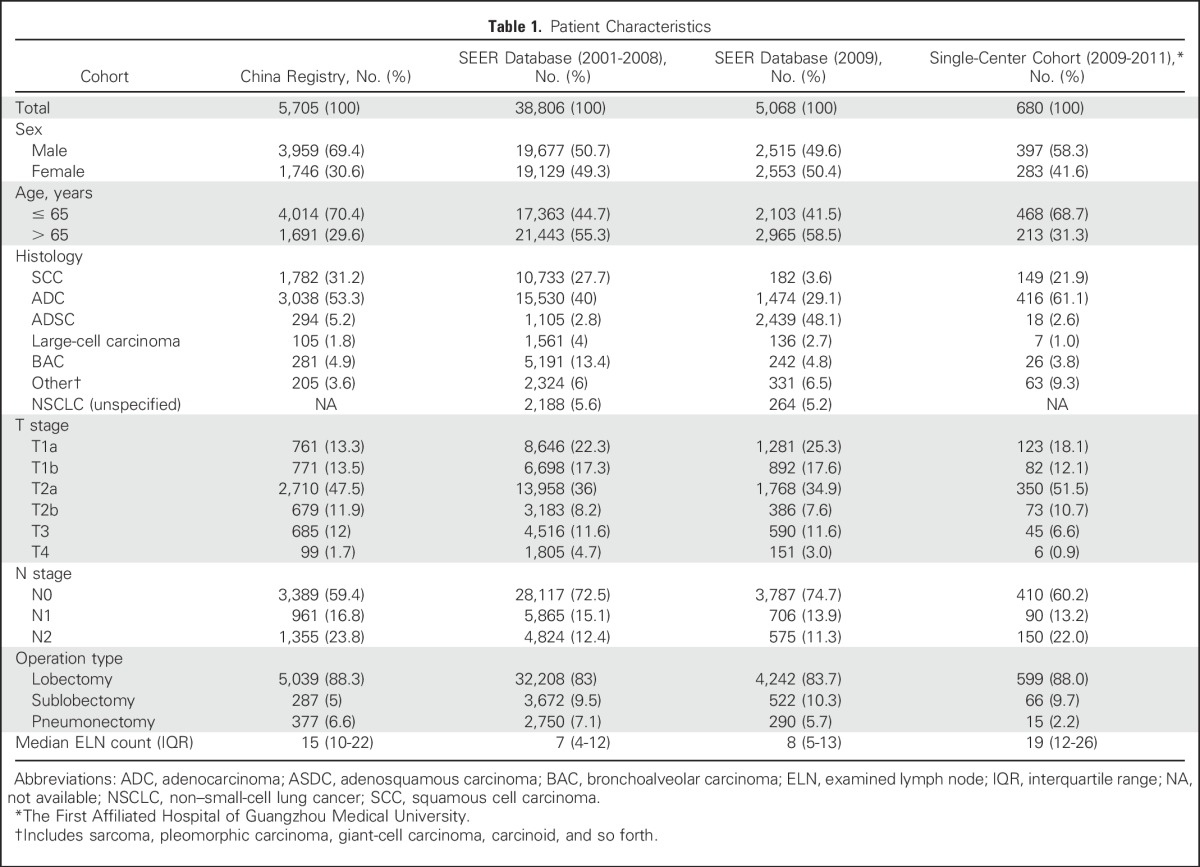
Patient Characteristics

### Number of Examined LNs and Population Stage Migration

The mean ELNs differed significantly within subcategories of T staging, N staging, histology, tumor location, and operation type in both cohorts (data not shown). All these factors were included in the regression model. Both cohorts showed a significantly proportional increase in N stage (from N0 to N1 and N2), with an increasing ELN count after adjusting for histology, T stage, tumor location, and operation type (SEER: OR, 1.038; 95% CI, 1.035 to 1.041; *P* < .001; China: OR, 1.012; 95% CI, 1.006 to 1.019; *P* < .001; Table 2). With regard to the correlation between examined LNs and positive LNs identified, a greater ELN count was associated with a greater number of positive LNs (linear regression, *R*^2^ = 0.917; *P* < .001), particularly in patients with node-positive disease (*R*^2^ = 0.915; *P* < .001).

**Table 2.**
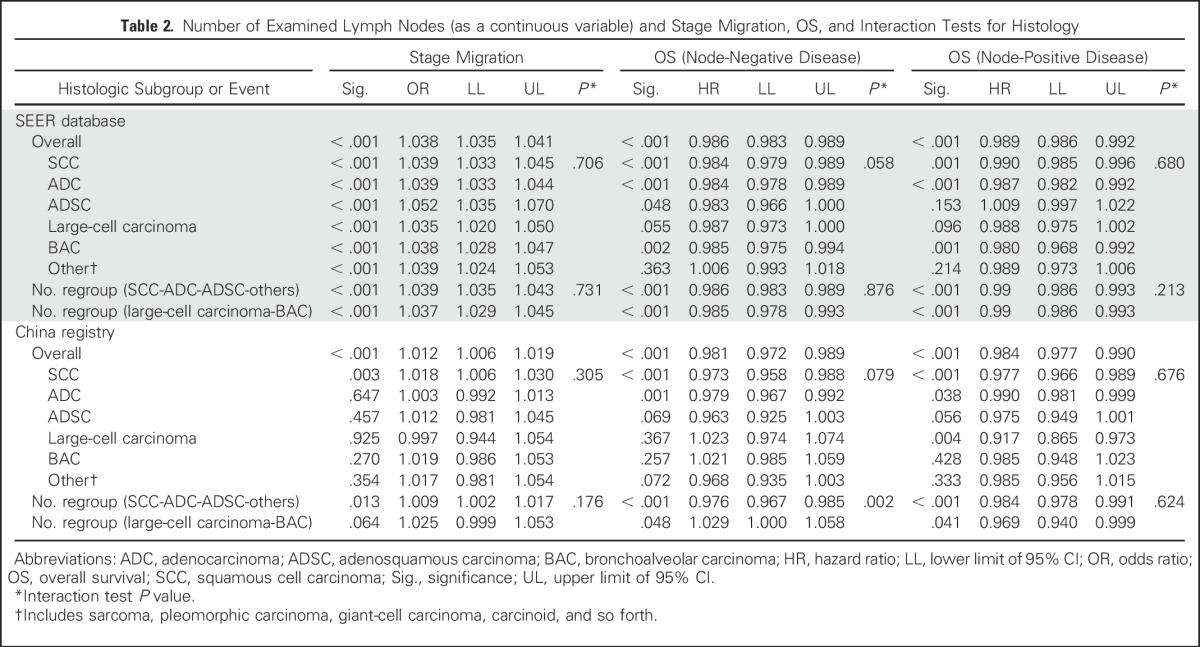
Number of Examined Lymph Nodes (as a continuous variable) and Stage Migration, OS, and Interaction Tests for Histology

The SEER cohort was also used to estimate the empirical distributions of the number of positive LNs; these results were then used to calculate the probabilities of having more positive nodes than observed (Appendix Table A1, online only). Special attention was paid to the probability of having at least one or more undetected positive nodes in patients who were considered to have node-negative disease. As expected, a greater number of ELNs correlated with a lower probability of stage migration.

### Number of ELNs and OS

After controlling for other prognostic factors (sex, age, pathology, T stage, and operation type), a greater number of ELNs was positively correlated with better OS among both patients with node-negative (N0) disease (SEER: HR, 0.986; 95% CI, 0.983 to 0.989; *P* < .001; China: HR, 0.981; 95% CI, 0.972 to 0.989; *P* < .001) and patients with node-positive (N1 and N2) disease (SEER: HR, 0.989; 95% CI, 0.986 to 0.992; *P* < .001; China: HR, 0.984; 95% CI, 0.977 to 0.990; *P* < .001), with a similar reduction in risk of death risk (Table 2). Of note, among patients with node-positive disease, the ELN count remained significant after adding the number of positive LNs to the Cox proportional hazards regression model (SEER: HR, 0.978; 95% CI, 0.975 to 0.982; *P* < .001; China: HR, 0.984; 95% CI, 0.977 to 0.990; *P* < .001). A consistent trend was observed in patients with N2 disease (SEER: HR, 0.993; 95% CI, 0.989 to 0.997; *P* = .002; China: HR, 0.985; 95% CI, 0.977 to 0.993; *P* < .001).

### Interaction Tests for Histologic Subgroups

By including the product of the value of histologic subgroup and ELN count, we tested the subgroup differences among various histologic types. In both regression models that included stage migration and OS, no significant subgroup difference was detected (Table 2). In addition, when bronchoalveolar carcinoma (an entity used in the pathologic classification of NSCLC during the data collection) and large-cell carcinoma (which was considered to be less affected by ELN count) were grouped together as one category, no subgroup differences between this combined category and the combined category that comprised of remaining histologic types were found except for OS in Chinese registry patients with node-negative disease. These results did not support a separate study of ELNs and OS correlation on the basis of histology.

### Cut Point Analysis for Patients With Node-Negative Disease and Validation

**Fig 2.**
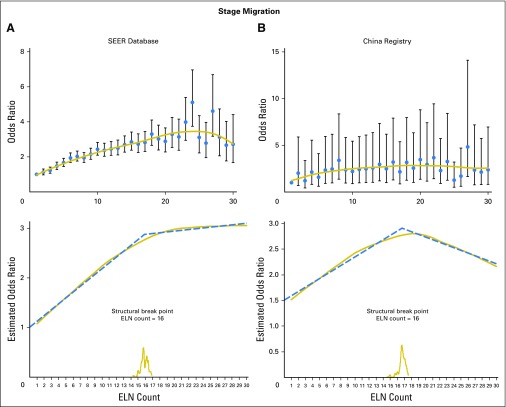

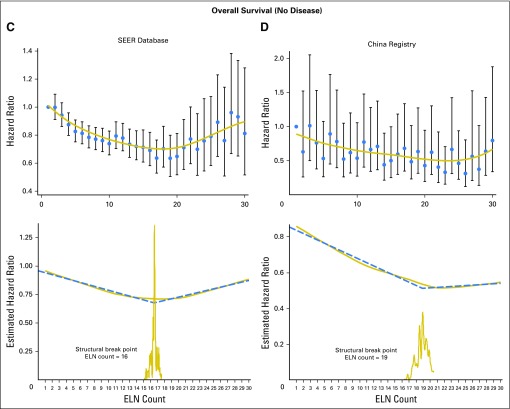
LOWESS smoother fitting curves of stage migration and overall survival and determination of structural break points with use of the Chow test. The fitting bandwidth was 2/3. (A) and (B) Stage migration was estimated by logistic regression after adjusting for T staging, N staging, histology, tumor location, and operation type in both cohorts. (C) and (D) Overall survival was estimated by using the Cox proportional hazards regression model after adjusting for sex, age, T staging, histology, and operation type. ELN, examined lymph node.

**Fig A1.**
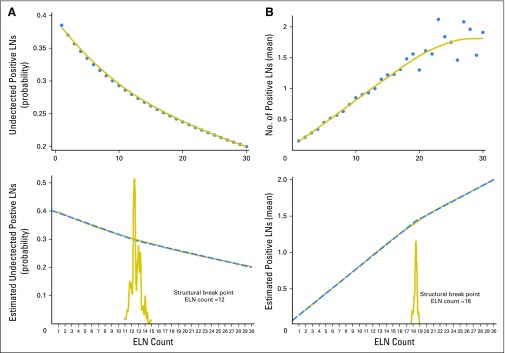
(A) LOWESS smoother fitting curves of estimated probabilities of undetected positive lymph nodes (LNs) in patients with declared node-negative disease. (B) Mean number of positive LNs as well as determination of structural break points by Chow test (fitting bandwidth = 2/3). ELN, examined lymph node.

Figure 2 shows the fitting curves and corresponding structural break points for both the OR of stage migration and the HR of OS in node-negative disease. The structural break points of the estimated probabilities of having positive nodes in patients with node-negative disease and the mean positive LNs identified at each ELN count were also determined (Appendix Fig A1, online only).

All the break points essentially were in agreement with one another (varied from 12 to 18). Because survival is the most important issue, we selected the structural break point of survival as the cut point. For generalizability and representativeness, we used a cutoff of 16 ELNs, which was generated from the SEER database.

**Fig 3.**
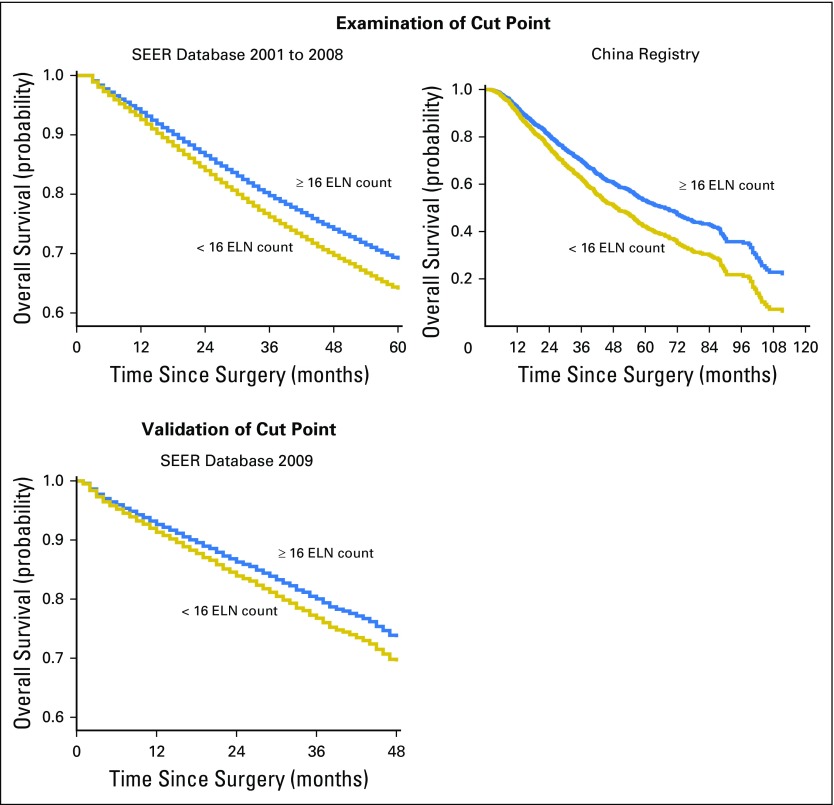
Stratification of overall survival among patients with node-negative non–small-cell lung cancer at the cut point of the number of harvested lymph nodes (16) on the basis of multivariable adjustments (other covariates were sex, age, T staging, histology, and operation type). ELN, examined lymph node.

**Fig A2.**
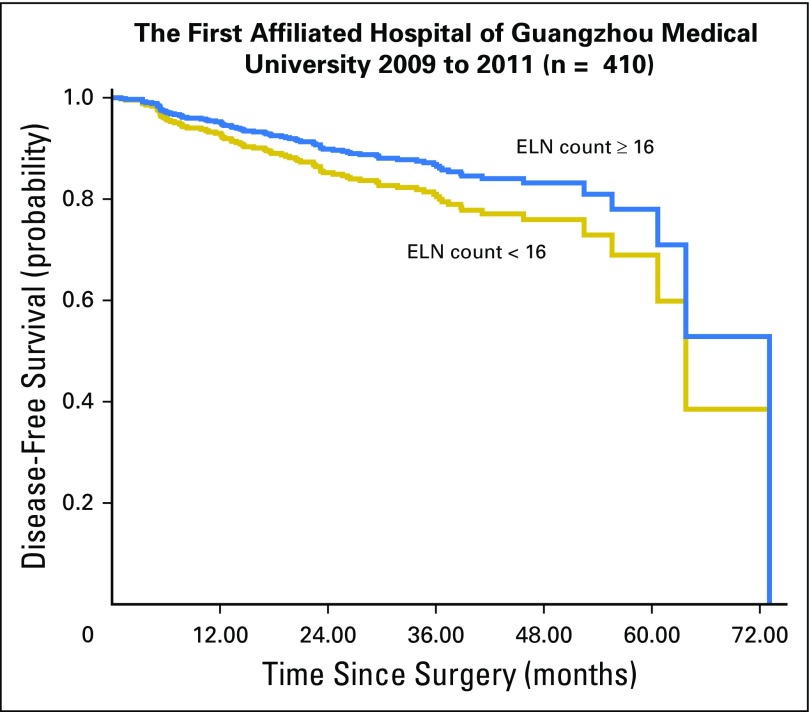
Stratification of disease-free survival among patients with node-negative non–small-cell lung cancer at the cut point of the number of harvested lymph nodes (16) on the basis of multivariable adjustments (other covariates: sex, age, T staging, histology, and operation type) of the single-center cohort (The First Affiliated Hospital of Guangzhou Medical University).

The cut point was first examined in both the Chinese and the SEER cohorts where it was derived. Survival analysis confirmed significantly reduced all-cause mortality of patients with at least 16 LNs harvested in node-negative NSCLC (SEER 2001 to 2008: HR, 0.830; 95% CI, 0.779 to 0.885; *P* < .001; Chinese registry: HR, 0.738; 95% CI, 0.655 to 0.831; *P* < .001) after adjusting for other prognostic factors (Fig 3). The cut point was then validated in the independent SEER 2009 cohort (HR, 0.837; 95% CI, 0.704 to 0.994; *P* = .043). Another independent validation from our single center (The First Affiliated Hospital of Guangzhou Medical University, 2009 to 2011) that used disease-free survival as a measurement was also performed (HR, 0.681; 95% CI, 0.435 to 1.066; *P* = .093; Appendix Fig A2, online only).

## DISCUSSION

In the current study, the stage migration analyses suggested that a larger number of LNs sampled was associated with a higher proportion of more-advanced N stage cases in the entire population, after adjusting for other risk factors for LN involvement. This association was confirmed by the trend of the mean number of positive LNs as well as the probability of accuracy of negative LNs with the mathematical model that was based on Bayes theorem. As illustrated, the examination of more LNs can reduce the risk of undetected positive LNs, which may result in a more thorough elimination of remnants and proper delivery of adjuvant chemotherapy to improve long-term survival. Accordingly, all cohorts exhibited consistent positive correlation between a greater number of ELNs and better OS in NSCLC with both positive and negative node status. With regard to histology, we found a highly significant positive correlation between ELN and OS in squamous cell carcinoma and adenocarcinoma; however, for other histologic types, the correlation was less uniform across cohorts most likely as a result of the paucity of cases or because the node status did not affect prognosis and treatment. However, interaction tests revealed still-insufficient power to study these issues separately on the basis of histology.

Several possible underlying reasons for the correlation between the number of ELNs and OS exist. First, patients with declared node-negative disease and with fewer ELNs may include some who actually have N1 or N2 disease. For patients with node-positive disease, those with a greater number of sampled LNs might include some who have received proper delivery of adjuvant chemotherapy as a result of the correct staging (eg, prevention of patients with node-positive disease from receiving a node-negative diagnosis), which would therefore improve OS. We also found hints of the benefits of clearance of a potential malignancy by dissecting more LNs. A greater number of ELNs was associated with improved survival in patients with resectable N2 disease among whom no stage migration would occur (patients with N3 disease were excluded). In this case, a higher ELN count means a smaller chance of undiscovered positive LNs (namely malignancy remnants, the potential source of recurrence), which thus indicates a more favorable outcome.

Another key issue is an adequate threshold of ELN count to allow a confident postoperative claim of node-negative disease. Patients with fewer ELNs than the threshold might have a higher risk of residual positive LNs and poorer survival. So far, recommendations on ELN count have not been made in the NCCN guidelines for NSCLC, but some studies have attempted to determine a benchmark, with suggestions ranging between 10 and 18 LNs for the disease overall.^14,18,27-30^ In the current study, we identified an optimal cutoff of 16 ELNs for node-negative NSCLC, which was validated in all cohorts. Of note, a study of complete hilar and mediastinal lymphadenectomy found that the mean total number of harvested LNs was 17.4 ± 7.3,^31^ which precisely agrees with the cut point we found. The threshold could be considered one of the reference indexes for defining inadequate LN sampling. We also observed that ELN count has a prognostic impact despite the number of positive nodes among patients with node-positive disease. However, finding more positive LNs would not change the treatment strategy because adjuvant chemotherapy is given routinely to patients with node-positive disease who can tolerate it. We believe that the positive LN ratio (positive LNs/ELNs) would be a better prognostic stratification tool for node-positive disease; therefore, we did not develop a cut point for ELN count.

To our knowledge, this study is currently the largest on such issues to use multicenter, real-world data sets with robust statistics. We sought to emphasize two major points. First, ELN count is associated with improved outcomes in NSCLC; therefore, surgeons and pathologists should pursue a maximal effort to explore the LNs. Second, an increasing need exists to set up a minimal number or range for evaluating the completeness of LN sampling for NSCLC. For example, the NCCN guidelines suggest that patients with node-negative disease and high-risk features receive adjuvant chemotherapy; one of these features is incomplete LN sampling. The number of LNs harvested could be one of the evaluation criteria. This study was based on real-world patient data; therefore, the surgical procedures, assessments, and enumeration of LNs varied among regions, surgeons, laboratories, and pathologists. This variance places a greater emphasis on adequate LN sampling and dissection by surgeons in clinical practice and on the careful exploration of the pulmonary LNs.

This study is limited by its retrospective nature. We were not able to investigate some other important points such as the respective impact of the number of N1 and N2 station LNs. We were unable to use uniform counting methods because of the population study design. In addition, two possible biases could have resulted in a miscount of LN number: underestimation as a result of the difficulty in separating each LN in the dissected tissues and overestimation because of fragmentation of nodal tissues during the removal of LNs, which might limit the application of a cut point. We conservatively suggest that only complete LNs be counted if LN fragmentation exists.

In conclusion, a greater number of ELNs is associated with more-accurate node staging and better long-term survival of resected NSCLC. We recommend 16 ELNs as the cut point for the evaluation of the quality of postoperative LN examination or prognostic stratification for patients with declared node-negative disease.

## Figures and Tables

**Table A1.**
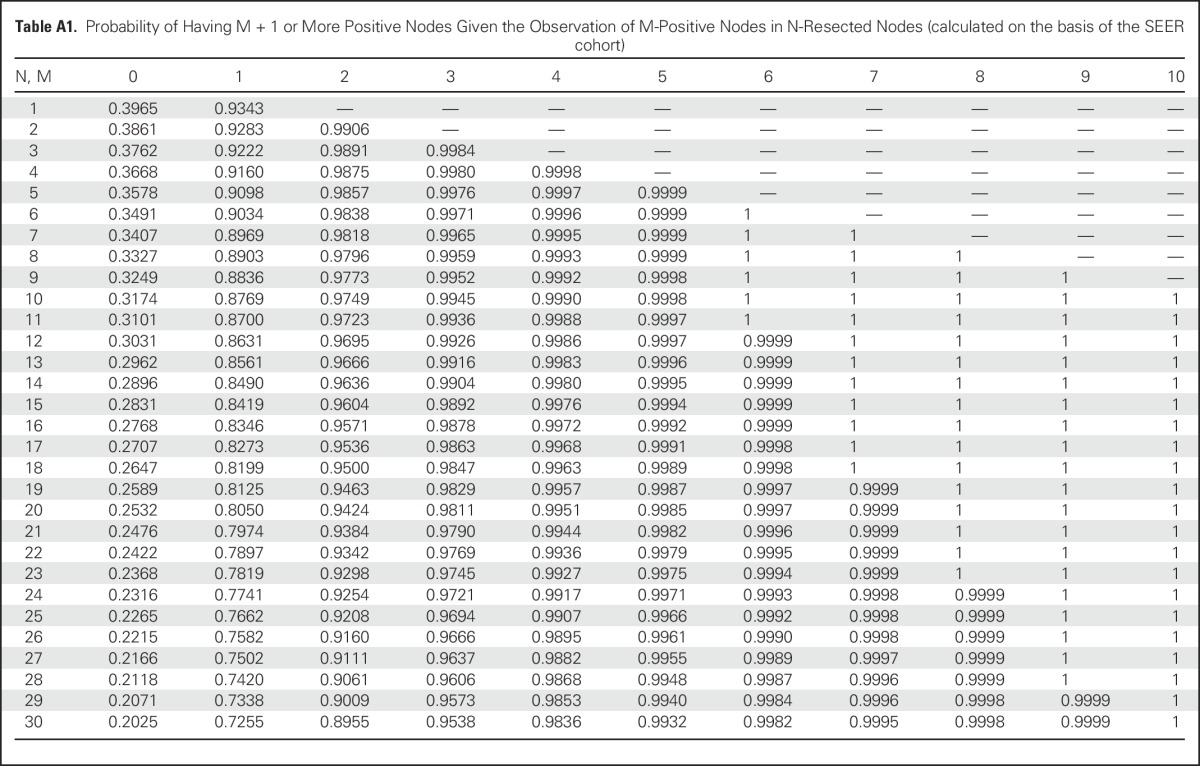
Probability of Having M + 1 or More Positive Nodes Given the Observation of M-Positive Nodes in N-Resected Nodes (calculated on the basis of the SEER cohort)
